# Supplementary figures and images for: Semiautomated generation of species-specific training data from large, unlabeled acoustic datasets for deep supervised birdsong isolation
Source: PeerJ. 2024 Sep 23;12:e17854. doi: 10.7717/peerj.17854 (PMC11426315; doi:10.7717/peerj.17854)

(A) Original

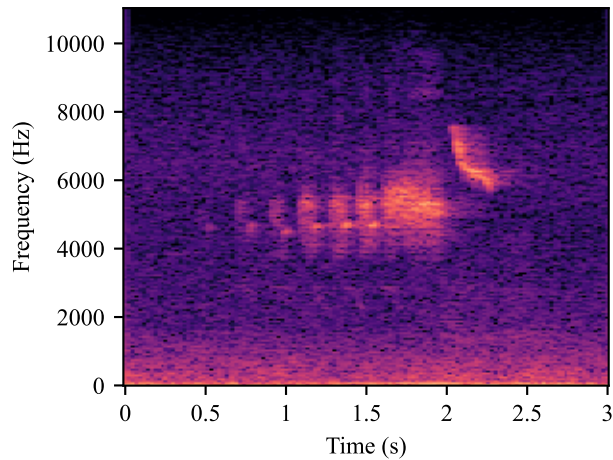

(B) After High Pass

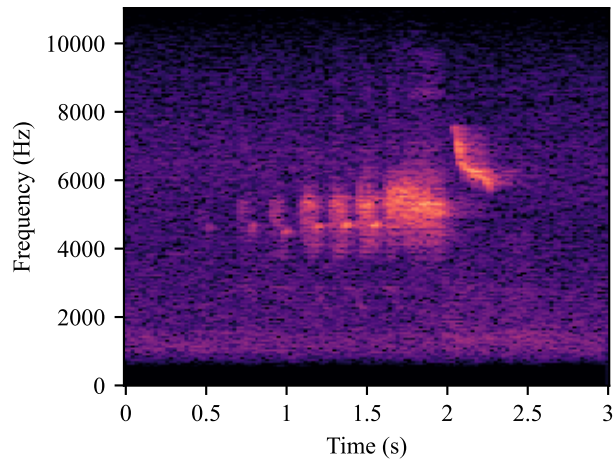

(C) After Normalization

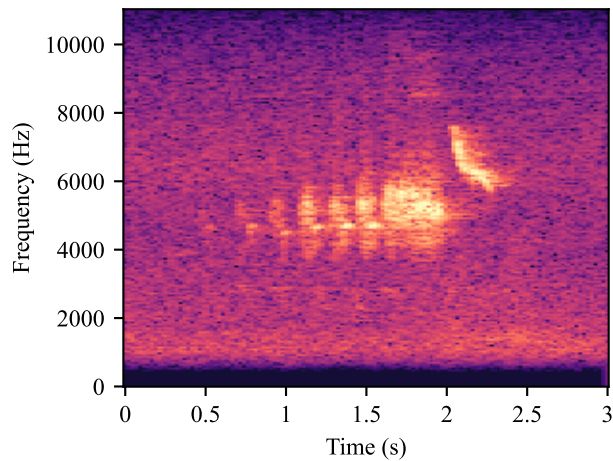

(D) After Noise Reduction

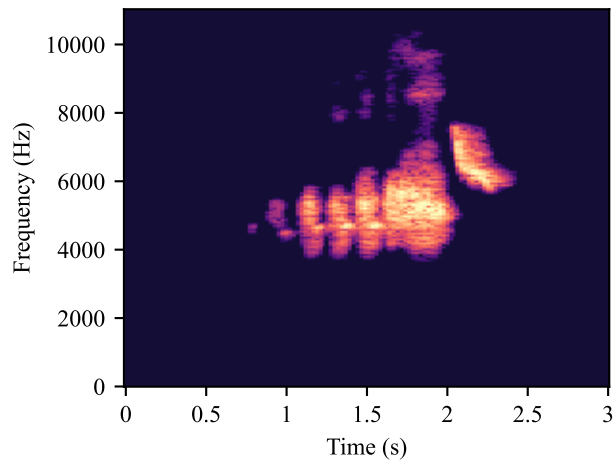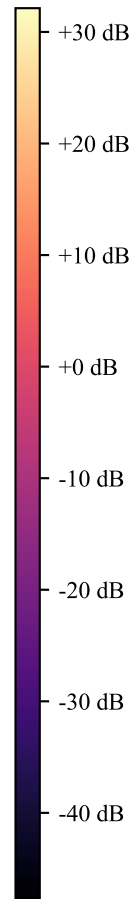

Supplement: Supplemental Information 1 — Our training dataset generation process involves extracting and cleaning potential birdsongs of interest from the full, passively-collected dataset. (A) Here, we first isolated a recording of a Golden-Cheeked Warbler (GCW) A song extracted from the raw recordings because it had a high joint probability of both containing a GCW birdsong and not containing any other bird or sound. (B) The removal of low-frequency road noise using high-pass filtering. (C) A volume increase after normalization. This standardizes the audio levels across recordings. (D) The elimination of most noise after spectral gating noise reduction. This results in a mostly clean birdsong that can be used to generate training examples. [file peerj-12-17854-s001.pdf]
